# Emerging Pathways to Non-Invasive Diagnosis in Endometriosis: Integrating Machine Learning, Deep Learning and Multi-Omics Biomarkers

**DOI:** 10.3390/diagnostics16121823

**Published:** 2026-06-12

**Authors:** Daniel Markov, Jasmin Gurung, Usman Khalid, Kristian Bechev, Vladimir Aleksiev, Galabin Markov, Elena Poryazova

**Affiliations:** 1 Department of General and Clinical Pathology, Medical University of Plovdiv, 4002 Plovdiv, Bulgaria; daniel.markov@mu-plovdiv.bg (D.M.); eporiazova@abv.bg (E.P.); 2 Department of Clinical Pathology, UMHAT “Pulmed”, 4002 Plovdiv, Bulgaria; 3 Faculty of Medicine, Medical University of Plovdiv, 4002 Plovdiv, Bulgaria; jasmingurung12@gmail.com (J.G.); usmankhalid957@gmail.com (U.K.); gabi_markov@abv.bg (G.M.); 4 Neurological Surgery, Pulmed University Hospital, 4000 Plovdiv, Bulgaria; 5 Department of Thoracic Surgery, UMHAT “Kaspela”, 4002 Plovdiv, Bulgaria; vl_alex@abv.bg; 6 Department of Cardiovascular Surgery, Medical University of Plovdiv, 4002 Plovdiv, Bulgaria; 7 Clinical and Experimental Morphology Division, Research Institute at Medical University of Plovdiv, Medical University of Plovdiv, 4002 Plovdiv, Bulgaria

**Keywords:** endometriosis, artificial intelligence, multi-omics biomarkers, machine learning, non-invasive diagnosis, deep learning imaging

## Abstract

Endometriosis is a chronic, debilitating condition affecting approximately 10–15% of reproductive-aged women and it is often associated with significant diagnostic delays due to its heterogeneity and unreliable non-invasive tests. Artificial intelligence (AI) offers innovative methods for improving endometriosis diagnosis, prognosis and research via advanced pattern recognition and data analysis capabilities. The integration of AI in diagnostic workflow has the potential to improve efficiency, accuracy, and patient outcomes. This review summarises current developments of AI—including machine learning, deep learning, and natural language processing—in the diagnostic workflow of endometriosis. It analyses different fields of diagnostics ranging from AI-assisted imaging in detection of pouch of Douglas to multi-omics biomarkers assisting the clinical decision process. AI can enhance accuracy, reducing diagnostic delays and supporting personalised treatment planning. However, there are multiple limitations, such as small datasets, overfitting, and lack of external validation and variability. Further research and evaluation are required before it can be implemented into healthcare systems. AI holds promise as a non-invasive, scalable adjunct to current diagnostics, potentially reducing the economic and personal burden endometriosis carries.

## 1. Introduction

Endometriosis is a multifaceted disease that is classed as a chronic, debilitating condition. It has a significant clinical impact, affecting approximately 1 in 10 women globally [1]. Endometriosis comprises three distinct phenotypes: superficial endometriosis, endometriomas, and deep endometriosis. According to the severity, amount, location, depth, and size of growth, it is divided into four stages, ranging from minimal, mild, moderate, and severe disease. Regardless of this classification, the prognosis of clinical outcomes and symptomatology remains ineffective [2]. It is a chronic condition, characterised by chronic abdominal pain and/or chronic pelvic pain (CPP). Endometriosis is diagnosed via a surgical procedure that locates the endometrium outside of the uterus [3]. Studies have shown that more than 60% of women diagnosed with endometriosis report CPP, and these patients are 13 times more likely to experience abdominal pain than healthy individuals. Despite this, endometriosis displays an array of non-specific symptoms ranging from gynaecological and gastrointestinal conditions [4]. Studies reveal an average diagnostic delay of 11.7 years in the USA and 8 years in the UK. Furthermore, the delay in diagnosis is greater in women reporting pelvic pain in comparison to infertility, implying that pelvic symptoms require more attention and consideration [5,6]. Despite the similarities in symptoms, the disease remains poorly understood, and current studies demonstrate no correlation between the extent of disease and symptomatology. Multiple ongoing research projects are currently in the works due to the poor efficacy of hormonal therapy. Endometriosis is one of the most common non-cancerous gynaecological proliferations in premenopausal women, affecting 10–15% of reproductive-aged women. Medical professionals face difficulties in diagnosis and treatment [7].

The evolving role of artificial intelligence (AI) can be observed in all aspects of life, especially in the health sector. Modern technologies have facilitated the growth of different AI-assisted strategies in healthcare. The digital revolution catalyses the progress of AI in medicine with new possibilities [8]. Reflecting its broad utility, the implications of AI can be visualised in the field of Obstetrics and Gynaecology, extending from foetal monitoring to reproductive medicine. Given the versatility and effectiveness, it holds substantial promise in improving the diagnostic workflow of endometriosis. Although there are publications that separately consider the application of artificial intelligence in sonographic diagnosis or in the analysis of biomarkers for endometriosis, there is a lack of comprehensive work integrating these approaches and critically evaluating their help for clinical practice. The present article seeks to fill this gap by comparing traditional diagnostic steps with the latest AI-assisted methods and pointing out both their advantages and limitations in diagnosis, prognosis and research, to help further the knowledge in endometriosis and consequently overcome the difficulties clinicians face and the devastating impact it carries [9]. By integrating imaging, molecular biomarkers, histopathology and large language model applications, this review provides a multidisciplinary perspective intended to support both clinical interpretation and computational development in endometriosis diagnosis.

## 2. Materials and Methods

A comprehensive literature search was conducted following the PRISMA guidelines (Supplementary Materials File S1) for scoping reviews to identify studies evaluating the application of artificial intelligence (AI) in the diagnostic workflow of endometriosis. The search was performed in Medline (via PubMed) and Google Scholar, covering publications from database inception to August 2025. The search strategy used combinations of the following keywords and Boolean operators: (“artificial intelligence” OR “machine learning” OR “deep learning” OR “neural networks” OR “natural language processing”) AND (“endometriosis” OR “deep endometriosis” OR “endometrioma” OR “pelvic pain”) AND (“diagnosis” OR “detection” OR “classification” OR “biomarker” OR “multi-omics” OR “ultrasound” OR “MRI” OR “histopathology”).

Additional manual searches were performed using the reference lists of relevant reviews and primary studies to capture articles not indexed in databases. Only peer-reviewed, full-text articles written in English were included. Conference abstracts, preprints, editorials, commentaries, and animal studies were excluded.

Screening and Selection: After removal of 75 duplicate records, 396 records were screened by two independent reviewers (J.G. and U.K.) based on titles and abstracts. Records were excluded at title and abstract screening if they were not related to AI-assisted endometriosis diagnosis, did not address diagnostic applications, or did not meet the predefined inclusion criteria. Full texts of potentially relevant papers were assessed for eligibility. Disagreements were resolved by consensus or consultation with a senior reviewer (D.M.).

Eligibility criteria:

- Inclusion: Studies reporting the use of AI-based methods for detection, diagnosis, classification, prediction, or biomarker identification in endometriosis (including imaging, molecular, histopathology, or language-based tools).
- Exclusion: Studies unrelated to diagnostic use (e.g., AI in treatment prediction only), non-original data, or insufficient methodological detail.

Data Extraction and Synthesis:

For each included study, the following variables were extracted: author, year, country, study design, population size, input modality (TVUS, MRI, histopathology, serum/omics, text data), AI technique or algorithm used, validation method, and diagnostic performance metrics (sensitivity, specificity, AUC).

Studies were grouped into four thematic domains for structured synthesis:

(1) Symptom-based and risk prediction models;
(2) AI-assisted imaging (TVUS/MRI);
(3) Histopathology and digital tissue analysis;
(4) Molecular and multi-omics biomarkers.

Within each domain, study findings were qualitatively summarised, and methodological consistency and validation approaches were compared.

**Figure 1:**
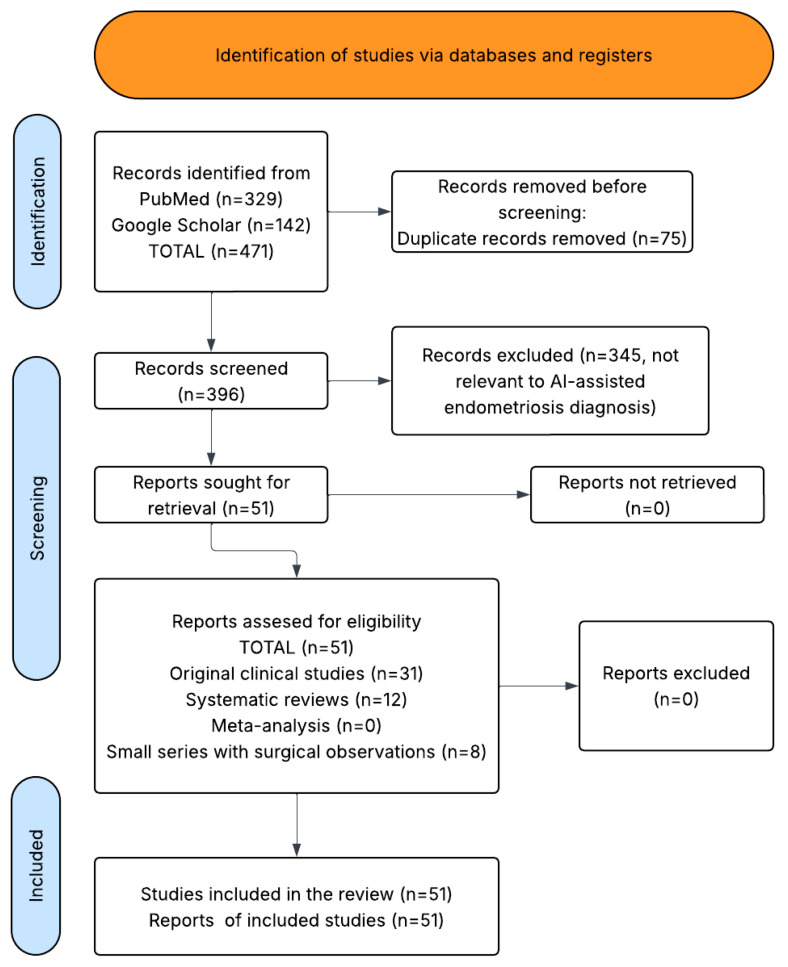
PRISMA flowchart summarising the study selection process, including records identified, duplicates removed, records screened, reports assessed for eligibility and final studies included in the review.

Quality and Reproducibility Assessment: Given the heterogeneity of the included studies, formal meta-analysis was not performed. Methodological quality was assessed qualitatively using predefined domains relevant to the study type. Diagnostic accuracy studies were assessed using QUADAS-2-based criteria, including patient selection, index test, reference standard, flow and timing, validation strategy and risk of bias. Review articles were considered using AMSTAR-2-based criteria where applicable, including clarity of research question, search strategy, study selection and synthesis approach. Additional reproducibility factors were recorded for AI-based studies, including dataset size, input modality, algorithm type, internal or external validation, reported performance metrics and clinical applicability (Figure 1).

## 3. Results

### 3.1. Artificial Intelligence

AI refers to a diverse range of applications used across various fields. Machine learning (ML) is an integral component of AI, which teaches computers to complete tasks without a predetermined specific programming of such tasks. It undergoes continuous enhancements via increased exposure to more data. This enables the systems to observe patterns and similarities by recognising objects from texts, images, or even acoustic data and extracting the information required [10]. Deep learning (DL) is a branch of ML that is difficult to comprehend, as it does not require any human input and it learns using sophisticated algorithms known as artificial neural networks. These models are particularly useful for analysing complex medical data, such as ultrasound images or histopathology slides, where manual interpretation may be time-consuming or subjective [11]. Numerous forms of AI are available, consisting of natural language processing (NLP) and large language models (LLM), which are AI systems trained on large volumes of text to understand and generate human-like language. Both forms are capable of understanding human language and generating human-like language [12]. AI could help decrease the major diagnostic delay in endometriosis [9].

Popular LLMs, such as Chat Generative Pre-trained Transformer (GPT) and other medically oriented chatbot systems, have also been explored in relation to endometriosis information delivery [13]. Their main potential role is currently supportive, particularly in improving patient education, awareness and access to general medical information [12,14]. More broadly, LLMs have shown potential in general medical question-answering and diagnostic reasoning tasks, including complex clinical cases and medical licencing-style examinations [15,16]. However, their diagnostic value remains indirect and limited. Studies assessing chatbot responses to endometriosis-related questions have reported variable accuracy, especially when answers are compared with guideline-based recommendations [17,18]. Therefore, LLMs should not be considered validated diagnostic tools for endometriosis at present, but rather adjunctive educational technologies requiring further evaluation before clinical integration (Table 1 and Table 2).

**Table 1:** Representative overview of selected emerging AI-related topics in endometriosis diagnosis and research. The table highlights key examples across symptom-based prediction, imaging analysis and molecular biomarker studies rather than listing all included studies.

Topics of Investigation	Study	Methodology	Clinical Utility
Symptom-based prediction	Bendifallah et al. [19]	Application of machine learning algorithm trained on 16 clinical and patient-reported symptom features to diagnose cases of endometriosis.	Early screening tool
Symptom-based predictive model	Nnoaham et al. [20]	Prospective, observational, two-phase multicentre study utilising 25-item questionnaire prior to surgery.	Help in early referrals for laparoscopy and prioritisation of surgical resources
Symptom-based diagnostic tool	Nnoaham et al. [21]	Integrated two multicentre studies from the GSWH and WHSS to develop and validate predictive models.	Early diagnosis and management
Assess automatic diagnosis of POD obliteration using TVUS	Macais et al. [22]	Two expert sonologists recorded TVUS videos dividing them into: training, validation and testing datasets and were assessed using pre-trained neural network	Non-invasive diagnosis of POD obliteration
Enhance POD obliteration from MRI by extracting diagnostic features from ultrasound via unpaired data	Zhang et al. [23]	A teacher model was created utilising TVUS data, and a student model was pre-trained with 3D masked auto-encoder, then fine-tuned using knowledge distillation.	Improving MRI based non-invasive diagnosis when ultrasound is unavailable
Non-invasive blood-based diagnostic model using panel of nine genes	Su et al. [24]	A nine-gene panel was created to differentiate patients with endometriosis from healthy individuals	Earlier non-invasive diagnosis using peripheral blood testing
Non-invasive diagnostic tool	Moustafa et al. [25]	Validate a panel of serum microRNAs from 100 symptomatic women undergoing gynaecologic surgery	Very accurate, minimally invasive serum-based diagnostics which can be applied preoperatively
Non-invasive biomarkers; persistent organic pollutants (POP)	Matta et al. [26]	Use ML algorithms to identify key POP biomarkers linked to deep endometriosis	Future biomarker screening for helpful diagnosis in endometriosis
Non-invasive biomarkers; plasma cytokines	Knific et al. [27]	Investigate whether plasma cytokine can differentiate women with endometriosis from controls using ML classifiers	Future alternative biomarker source for non-invasive diagnosis
Non-invasive biomarkers; programmed cell-death-related genes	Xie et al. [28]	Identify programmed cell-death-related gene biomarkers using bioinformatics and ML approaches	Non-invasive, multi-omics gene targets holding potential for diagnostics

**Table 2:** Comparative summary of selected AI-based diagnostic studies in endometriosis, including AI application, dataset or modality, validation strategy, reported performance metrics and clinical applicability or limitations.

Study	AI Application	Dataset/Modality	Validation	Performance	Clinical Applicability/ Limitation
Bendifallah et al. [19]	Symptom-based ML screening	1126 patients, 608 controls, clinical and symptom features	Training and validation sets	Sensitivity 0.82–1.00, specificity 0–0.80, AUC 0.50–0.89	Potential screening tool, but variable specificity and external validation required
Nnoaham et al. [20]	Symptom-based prediction model	Multicentre pre-laparoscopy cohort	Multicentre validation	AUC 68.3 for any-stage disease, AUC 84.9 for stage III/IV disease	More useful for advanced disease, limited accuracy for early-stage disease
Maicas et al. [22]	DL analysis of TVUS sliding sign	TVUS videos for POD obliteration	Training, validation and test sets	Accuracy 99.9%, specificity 90%	Promising for standardising TVUS interpretation, dependent on expert video acquisition
Zhang et al. [23,29]	Knowledge distillation from TVUS to MRI	TVUS and MRI datasets	Internal validation	MRI AUC improved from 65.0% to 90.6%	May improve MRI interpretation, but requires external/prospective validation
Guerriero et al. [30]	ML detection of rectosigmoid deep endometriosis	222 training cases, 110 test cases, ultrasound markers	Train/test split	Accuracy 73%, AUC 0.82	Moderate performance, may assist but cannot replace expert assessment
Bendifallah et al. [31]	Salivary miRNA ML model	Salivary miRNA profile	Cross-validation	Sensitivity 96.7%, specificity 100%, AUC 98.3%	Highly promising non-invasive test but needs independent validation
Su et al. [24]	Gene-expression diagnostic model	Tissue and blood transcriptomic datasets	Model validation datasets	AUC 0.920 tissue, AUC 0.942 blood	Potential blood-based diagnostic tool, needs prospective clinical validation
Moustafa et al. [25]	Serum miRNA classifier	100 symptomatic surgical patients	Surgical diagnosis reference	Classifier accuracy 0.94	Promising preoperative biomarker panel, limited by small sample size
Knific et al. [27]	Cytokine-based ML model	116 endometriosis patients, 94 controls	Internal testing	Sensitivity 40%, specificity 65%, AUC 0.61	Limited diagnostic value based on current evidence
Stegmann et al. [32]	Lesion prediction model	114 women, 487 lesions	Internal validation	Sensitivity 88.4%, specificity 24.6%	May support intraoperative assessment, but low specificity limits clinical utility

### 3.2. Enhanced Endometriosis Prediction

In clinical pathways, an immediate application of AI is to prioritise patients using history and symptom profiles, supporting earlier screening and referral decisions. Early work explored whether endometriosis could be predicted non-invasively using symptoms, clinical examination and ultrasound findings. In one study, clinical information was collected from 90 women before laparoscopy or laparotomy to build a classification tree. Ovarian endometriosis was correctly classified in 100% of cases using ultrasound and examination findings, with no false positives; however, only 38% of non-ovarian endometriosis was correctly classified [33].

Building on this symptom-based approach, Bendifallah et al. investigated ML algorithms using 16 clinical and patient-reported features, including patient history, demographics, phenotypes and treatment data. The model was developed using data from the Ziwig Health platform, which included 1126 patients with endometriosis and 608 controls [19]. Sensitivity ranged from 0.82 to 1.00, specificity from 0 to 0.80 and AUC from 0.50 to 0.89 across training and validation sets. This study suggests that an ML-based algorithm may be useful as a screening tool for healthcare practitioners and may support patient involvement in earlier recognition of symptoms and shared decision-making [19].

Similarly, Nnoaham et al. developed a symptom-based model to predict endometriosis before first laparoscopy. Prediction of any-stage endometriosis was limited, with an AUC of 68.3, whereas prediction of stage III/IV disease was stronger, with an AUC of 84.9, sensitivity of 75.8% and specificity of 75.8% [20].

Since the symptoms vary within different populations, two multicentre studies, the Global Study of Women’s Health (GSWH) and Women’s Health Symptom Survey (WHSS), were initiated to collect standardised, epidemiological data and develop a symptom-based diagnostic tool. A six-week pilot study conducted in Oxford, United Kingdom, showed endometriosis was found in 47.4% of 27 participants. These studies aim to develop a validated, symptom-based diagnostic tool that will prove beneficial across the globe [21]. Despite the heterogeneity of the disease, studies are ongoing to focus on clinical prediction models for successful surgery. For instance, the CRESCENDO (Creating Prediction Model to predict Surgical Success in Endometriosis). AI looks promising in identifying reliable predictors [34]. Multivariate logistic regression was utilised to predict the possibilities of voiding dysfunction post-surgery. Clinical and imaging features were used to create these models. Patient’s surgical plans could be tailored according to the individual risk [35]. Application of AI helps expand knowledge about predictors of successful treatments, improving the standard of care, while simultaneously respecting patient autonomy [31,35].

Building on these symptom- and risk-based prediction models, the next section focuses on imaging-based AI applications, which aim to enhance diagnostic accuracy and address delays caused by the limitations of current radiological expertise and workflows.

### 3.3. AI in Diagnosis

Once patients are triaged, imaging plays a crucial role in confirming diagnosis and defining disease extent. Diagnostic imaging delays contribute to misdiagnosis and treatment delays, as symptoms are also associated with other conditions. A study consisting of 11,793 patients demonstrated that 35.3% of patients experienced diagnostic delay of 3–5 years. These patients also suffered from pre-diagnosis endometriosis-related symptoms and required more healthcare services, resulting in higher costs [36]. The International Deep Endometriosis Analysis (IDEA) group is a consensus published in 2016 to help describe sonographic patterns of different phenotypes of endometriosis to encourage global consistency for descriptions of endometriosis ultrasound location and extent [37]. Using this consensus, AI-assisted models utilising subtypes of DL, referred to as transfer learning and deep neural networks, have been generated. Maicas et al. created Kinetics-400, applying a technique known as transfer learning (where knowledge from a large, general dataset is adapted to a new medical task) with a dataset of 306,245 videos showing multiple human actions. In transfer learning, the pre-trained models hold the ability to adapt to a new task where they can apply their knowledge to different domains of interest, which is particularly valuable in gynaecological imaging, where large annotated datasets are often unavailable. In this instance, transvaginal ultrasound (TVUS) videos were used. It is difficult to assess the exact relationship between the kinetics-400 dataset and the TVUS dataset, as this approach relies on complex neural network architectures that do not easily reveal the features being used, making direct interpretation challenging. However, the model demonstrated an accuracy of 88.8% with a specificity of 90.0% in categorising pouch of Douglas (POD) obliteration, validated by two expert ultrasound specialists. Although the IDEA group has recommended routine evaluation of POD obliteration, there are no radiology societies suggesting the same [22]. In another study implementing the IDEA consensus, Zhang et al. developed a knowledge distillation training method using TVUS and POD obliteration from MRI scans. The foundations were built upon TVUS due to its superiority in detecting POD obliteration. Subsequently, the student model was trained on MRI, which underwent fine-tuning, following a knowledge distillation approach (where a ‘teacher’ model guides a new model to learn efficiently from smaller datasets), improving the diagnostic area under the receiver operating characteristic curve from 65.0% to 90.6%. This study highlights the benefits of combining imaging datasets in AI algorithms and the potential it holds for future applications in endometriosis imaging signs and locations [23].

The ability to relay knowledge across TVUS and magnetic resonance imaging (MRI) is one of its advantages, which helps in non-invasive diagnosis of endometriosis by identification of POD obliteration [38]. Despite this advantage, there are insufficient clinicians with expertise in the diagnosis of image-based endometriosis. Additionally, most patients will not undergo both TVUS and MRI scans, and the detection of POD obliteration is far more challenging on MRI. To overcome this, Zhang et al. created a high-performance TVUS analysis model with an AUC of 96.9% as a mentor to train similar models on MRI images. This approach amplified the performance on MRI images from an AUC of 65% to 90.6% [29]. Another study examined the accuracy of TVUS and MRI in the identification of deep pelvic endometriosis; however, it required radiologists with more than 10 years of experience in gynaecological imaging studies to accomplish an accuracy of 88% on TVUS and 55% on MRI [38]. Seven different traditional ML models trained on the US “soft marker” for the prediction of rectosigmoid endometriosis were used on a training set of 222 people and a test set including 110 people. Neural Net demonstrated 73% accuracy and an AUC of 0.82 with similar results to the sigmoid model [30]. Currently, the technology may not be completely ready for automated image analysis, but the results look promising, providing hope for AI models to replicate human performance and aid in screening of patients with a high likelihood of endometriosis diagnosis, guiding them to correct referrals and further care [3].

Beyond imaging, tissue-level and molecular analyses offer a different layer of diagnostic information, providing opportunities for AI to support histopathological interpretation, biomarker identification, and a deeper understanding of disease heterogeneity.

### 3.4. Molecular Pathology

Histopathology remains the gold standard for confirming endometriosis, and recent advances are bringing AI into this domain. The diagnosis of endometriosis is confirmed by examination of biopsy samples stained with haematoxylin and eosin (H&E); however, the heterogeneity of endometriosis may produce false-negative results. Modern methods are being adopted to improve the detection of endometriosis, thereby enhancing the treatment decisions [39]. Recent developments in digital pathology create avenues for automatic detection, quantification, and stratification of cells within endometrial tissues. Utilising QuPath software, a widely used open-source tool for automated slide analysis, 26 tissue samples were extracted from patients undergoing laparoscopy for suspected endometriosis. Immunohistochemistry (IHC) was employed to detect cytokeratin (epithelial cells) and CD10 (stromal cells) markers.

Recent developments in digital pathology create opportunities for automated detection, quantification and stratification of cells within endometriosis tissue. Using QuPath, an open-source digital pathology platform, McKinnon et al. analysed 26 tissue samples from patients undergoing laparoscopy for suspected endometriosis. Immunohistochemistry was used to identify cytokeratin-positive epithelial cells and CD10-positive stromal cells. Through computer-assisted image analysis, the study quantified epithelial and stromal cell populations and reported greater stromal cell abundance in deeply infiltrating endometriosis compared with superficial peritoneal lesions (*p* = 0.0477), as well as a positive correlation between endometrial cell count and abdominal pain score (*p* = 0.0005) [40]. This demonstrates how digital image analysis can provide more objective and reproducible histological characterisation than manual assessment alone.

Similarly, Korman et al. developed an ML- and DL-based image-analysis pipeline for multiplex-stained endometrial tissue sections using a six-plex immunohistochemistry panel consisting of PanCK, CD10, calretinin, Ki67, DAPI and α-SMA. The AI-based approach enabled automated tissue segmentation and identification of epithelial, stromal and immune cell populations across individual slides, supporting objective and reproducible quantification beyond traditional manual microscopy [41].

B-cell lymphoma 6 (BCL6) was initially described in large B-cell lymphoma as a locus disrupted by chromosomal translocations, and it functions as a crucial regulator of both normal and cancerous lymphocyte biology. It orchestrates B-cell germinal centre (GC) formation, maintenance, suppresses DNA checkpoints, and promotes proliferation, therefore contributing to lymphomagenesis. Alongside B-cells, BCL6 is exclusively expressed in follicular helper T cells (TFH), where it serves as a lineage-defining transcription factor essential for TFH differentiation and maintenance [42]. Recent studies demonstrate the promise of BCL-6 as a diagnostic biomarker for the detection of endometriosis in women [42,43]. Previous studies employed a semi-quantitative scoring system known as HSCORE to analyse the IHC staining; however, it consisted of limitations, such as lack of objectivity, robustness, and reproducibility, impacting intra- and inter-observer variability. Squatrito et al. investigated the expression of BCL-6 in varying degrees of endometrial tissues of women and fertility status using both traditional and digital-assisted tools. The comparative analysis demonstrated that the HSCORE was significantly higher (3.625) compared to controls (2.095), achieving a *p* value of 0.0053. Regarding digital image analysis, infertile women with stage IV endometriosis displayed greater intensity of BCL6 expression, suggesting a higher and more heterogeneous expression. Furthermore, the positive correlation between HSCORE values and digital image analysis results validates the reliability of digital-assisted methods [44].

Beyond histopathology, multi-omics approaches, such as transcriptomics and miRNA profiling, are emerging as powerful tools for uncovering molecular signatures of endometriosis, with AI playing a central role in managing and interpreting these high-dimensional datasets.

### 3.5. Multi-Omics in Endometriosis

Molecular analyses, particularly miRNA-based diagnostics, are increasingly being investigated for their potential to complement imaging and histopathology. Novel studies relative to microRNA(miRNA) are currently under investigation for their potential role as a non-invasive diagnostic tool [45]. The use of saliva as a disease biomarker is a trending subject due to its stability, as it is not affected by coagulation. Salivary miRNAs are currently under examination to find specific miRNAs for endometriosis. The capability of AI to analyse a large quantity of datasets allow for augmenting of diagnostic processes. The Random Forest algorithm was deployed on 109 miRNAs. Following feature selection and cross-validation, the diagnostic miRNA signature obtained 96.7% sensitivity, 100% specificity, and an AUC of 98.3%. This signifies the potential for a readily available non-invasive tool reducing delay in diagnosis [31]. Su et al. utilised 10 binary classification algorithms to develop a nine-gene panel endometriosis messenger RNA score (EMScore) to differentiate patients with or without endometriosis. The results were promising, with the areas under the receiver operator characteristic curve of the diagnostic model being 0.920 for tissue samples and 0.942 for blood samples [24]. A prospective study consisting of 100 subjects demonstrated increased levels of 4 serum miRNAs: miR-125b-5p, miR-150-5p, miR-342-3p, and miR-451a. The receiver operating characteristic areas under the curve ranged from 0.68 to 0.92. Application of a classifier resulted in a higher accuracy score of 0.94. According to the American Society of Reproductive Medicine staging, all miRNAs could be differentiated in stages I/II from the control group and stage III/IV from the control group. Results also showed that the phase of the menstrual cycle or hormonal therapies had no significant effect on the expression of miRNAs. The brilliance of AI in diagnosing endometriosis through serum miRNA analysis holds great benefits, such as decreased time to diagnosis, surgical risk, disease progression, and the healthcare costs [25].

Integrating these molecular and omics-based findings with presurgical assessment methods represents the next step toward comprehensive, personalised diagnostic pathways for endometriosis (Table 3).

**Table 3:** Summary of molecular and multi-omics biomarkers investigated for AI-assisted endometriosis diagnosis, including biomarker category, specific biomarker or panel, sample type, analytical approach and diagnostic relevance.

Biomarker Category	Specific Biomarkers/Panel	Sample Type	AI/Analytical Approach	Diagnostic Relevance
Salivary miRNAs [31]	Selected diagnostic signature from 109 miRNAs	Saliva	Random Forest with feature selection and cross-validation	Non-invasive diagnotic signature with high reported sensitivity, specificity and AUC
Gene-expression panel [24]	Nine-gene EMScore panel	Tissue and blood	Ten binary classification algorithms	Differentiated endometriosis from controls with high AUC in tissue and blood samples
Serum miRNAs [25]	miR-125b-5p, miR-150-5p, miR-342-3p and miR-451a	Serum	miRNA classifier	Minimally invasive preoperative biomarker panel
Persistent organic pollutants [26]	Organochlorine persistent organic pollutant patterns	Biomarker/exposure dataset	Five ML models	Potential association with deep endometriosis and biomarker discovery
Plasma cytokines [27]	Plasma cytokine profiles	Plasma	ML classifiers	Limited diagnostic performance in current evidence
Programmed cell-death-related genes [28]	TNFSF12, PDK2 and AP3M1	Gene-expression/bioinformatic datasets	Mendelian randomisation and ML	Candidate molecular biomarkers for diagnosis and personalised treatment research

### 3.6. Presurgical and Molecular Diagnostics in Endometriosis

Presurgical and molecular diagnostic strategies aim to bridge the gap between early detection and definitive diagnosis, combining laboratory insights with clinical evaluation. Current laparoscopy with histological confirmation remains the ideal diagnostic and therapeutic choice; however, it does pose surgical risk. Extensive research for reliable biomarkers will be highly beneficial in treatment decisions [46]. Traditional linear and regression models may lead to unstable and unreliable results when variables are correlated. Application of innovative ML models may enhance epidemiological research to improve the results. In a retrospective case study, five AI models helped in the identification of connections between different organochlorine persistent organic pollutants. These algorithms demonstrate the ability of AI to handle complex data [26]. Various genes have been discovered to help explain the pathogenesis of endometriosis; however, functional validation of such vast data requires great effort. Using natural language processing of the PubMed database integrated with text mining of AI allows for candidate gene prioritisation. Among the 15,207 endometriosis-related genes, 724 genes were retrieved and ranked according to priority. MAPK1 was one of the top five genes with a very high score of 0.988 (measured on a score of 0 to 1). These could be valuable in the research of diagnostic biomarkers and therapeutics of endometriosis [47]. Other biomarkers are also being analysed for their involvement in endometriosis. As it is an inflammatory process, different cytokines were assessed for their relevance. In this study, ML algorithms were employed to validate the hypothesis that models trained on fewer proteins would yield better results. Additionally, the models also checked for any relationship between the 116 patients with endometriosis and the 94 controls. The results were unsatisfactory, showing a sensitivity of 40%, specificity of 65% and AUC of 0.61, exposing the limited potential of cytokines for the diagnosis of endometriosis [27]. Past studies have shown that patients with endometriosis demonstrate abnormal activation or inhibition of programmed cell death (PCD), leading to pathological cell death. To identify biomarkers involved in endometriosis, this study utilised Mendelian randomisation and machine learning algorithms. It concluded three essential biomarkers with upregulation of TNFSF12 and PDK2 genes, with downregulation of AP3M1. Not only does this prove useful for early diagnosis, but it also opens up new avenues for personalised treatment as molecular docking studies show increased binding affinity with current drugs in clinical practice [28].

The presurgical diagnostic methods increase the accuracy of endometriosis; however, ultrasound, MRI, and additional lab tests lack adequate sensitivity and specificity [32]. Based on the 2000 ACOG Practice Bulletin [46] and the 2005 ACOG Committee Opinion [48], it is acceptable to diagnose endometriosis based solely on visual findings at laparoscopy. Positive histological findings are not necessary for diagnosis in all cases. Rather, it is done to confirm the suspicions of the surgeon during surgery, and the decision lies in their hands [49]. Visual assessment shows intra-physician variability with high rates of misdiagnosis and poor correlation with the histologic findings [50,51]. Stegmann and colleagues created a model utilising lesion characteristics to aid in identifying high and low probability lesions. The data consisted of 114 women with complete data on 487 lesions and achieved a sensitivity of 88.4% and specificity of 24.6%. It correctly classified a lesion with 66.5% success [32].

## 4. Discussion

AI in endometriosis can gain valuable insights into the disease itself as well as the diagnostics. This could potentially help lessen the burden and the impact it carries in the healthcare system. The application of AI could be advantageous in the diagnosis, treatment, and follow-up of endometriosis if under the supervision of a gynaecologist. As this was one of the first to assess the knowledge of ChatGPT associated with endometriosis, more research is required, for example, public understanding of its answers, subjective answers, and the time frame of the study, as the internet is constantly updated with new information [17]. Even though the answers from LLMs are being evaluated by experts, they could be affected by the personal opinions of the experts. To mitigate bias, guidelines were integrated into the evaluation process. It is important to note that the 10 sample questions do not consist of what the patients might ask about endometriosis, and reading levels vary between patients; therefore, the structures of the questions may be different. Additionally, there are no validated models to assess the LLM answers [18].

### 4.1. Clinical Readiness and Comparative Performance

#### 4.1.1. Current AI Variability Across Domains

Across the different domains reviewed, including TVUS-based imaging, biomarker panels, molecular approaches, and chatbot evaluations, there is considerable variability in study design and methodological rigour. Many studies rely on retrospective data or convenience samples, often with limited sample sizes that reduce statistical power and external validity. Direct comparative analyses between modalities are scarce, making it difficult to determine their relative strengths and weaknesses. In several cases, internal validation methods were applied, but external validation and head-to-head comparisons are largely absent. These limitations should be considered when interpreting reported performance metrics, as they may overestimate real-world effectiveness.

#### 4.1.2. Symptom-Based Tools and Imaging

Non-invasive diagnostic tools based on symptoms, signs, and ultrasound findings have proven beneficial in ovarian endometriosis; however, a large proportion of women experience infertility and pain and therefore, these symptoms alone are not reliable [33]. Some of the studies were based on self-questionnaires, and over 50% of the 1140 patients did not complete the survey. Asking individuals if they had endometriosis could introduce bias as well. It is vital to note that approximately 20% of endometriosis patients may be asymptomatic, which could potentially invalidate the questions. The validation set also did not include symptomatic patients with normal clinical examination and MRI features [19]. To avoid overfitting of the small sample, the bootstrap method and cross-validation were carried out. Before translation into clinical practice, external validation is crucial, yet it was not applied in this study [20]. Current clinical standards in sonographic diagnostics rely heavily on expert-performed transvaginal ultrasound following the IDEA consensus, which provides a structured approach for assessing endometriosis location and extent. While this remains the gold standard for preoperative imaging, it is limited by operator dependency and variability in interpretation. Emerging AI-assisted approaches, particularly those using transfer learning and neural network models to analyse the sliding sign, aim to standardise image interpretation and improve detection of pouch of Douglas obliteration. These methods offer the potential to complement established TVUS protocols by enhancing diagnostic accuracy and reproducibility, especially in settings with limited specialist expertise. Utilisation of pre-existing data excludes the possibility of choices of data and matching of factors in development and external validation data sets [34]. Overfitting in AI is a common problem, and to overcome this, smaller training sets are used; however, this decreases its diagnostic performance with low variability in TVUS. To ensure good data, performing a sliding sign and correctly recording the video clip is essential for the model to study from. The sonologists should be well trained on the sliding sign, as the foundations of the AI are built upon this. Only one brand of ultrasound machine was used in this study. To diversify the results, different ultrasound machines, sonographers, and radiologists should be used. Additionally, the high rate of endometriosis could be related to the location, as this place specialises in gynaecology [22]. As these studies often originate from specialised centres with experienced operators and homogenous populations, their findings may not fully reflect real-world variability or performance in broader clinical settings. Larger, multicentre cohorts are therefore essential to confirm these early observations.

Comparison with guideline-based workflow: Conventional pathways prioritise clinician-performed TVUS per IDEA consensus, selective MRI, and clinical judgement to determine the need for laparoscopy. The AI-enabled pathway retains these steps but adds standardised video acquisition and algorithmic decision support at the TVUS stage, optional AI-assisted MRI interpretation, and selective use of validated non-invasive biomarkers to improve triage in equivocal cases. Thus, AI functions as an adjunct to reduce operator dependency, enhance reproducibility, and potentially shorten time to diagnosis, while remaining anchored to current guidelines.

#### 4.1.3. Clinical Readiness

Although AI applications in imaging modalities, such as TVUS and MRI, as well as biomarker-based approaches, are relatively well developed with measurable diagnostic performance and initial external validation, other areas remain less mature. NLP and large language models are still at a proof-of-concept stage, with limited evaluation of clinical integration or real-world reliability. Similarly, molecular diagnostic approaches, while promising, are restricted by small sample sizes, limited standardisation, and a lack of large-scale validation. This discrepancy highlights the varying readiness levels of different AI applications, with imaging and biomarker tools being closer to potential clinical translation, whereas NLP/LLMs and molecular diagnostics require further foundational work before routine clinical use can be envisaged.

### 4.2. Limitations of Current Evidence

While numerous proof-of-concept studies have demonstrated promising diagnostic performance, several interlinked factors continue to impede clinical translation. First, the predominance of small, single-centre datasets leads to model overfitting and limits external validity. Second, the absence of standardised data acquisition protocols, particularly for ultrasound and molecular biomarkers, hinders reproducibility and cross-centre comparisons. Third, the lack of multicentre external validation and head-to-head modality comparisons prevents robust benchmarking against existing clinical standards. Fourth, ethical and regulatory frameworks specific to AI in gynaecology remain underdeveloped, creating uncertainty around accountability and safety. Finally, clinician trust and workflow integration have not yet been systematically addressed. Together, these limitations explain why AI remains confined to research settings rather than routine diagnostic pathways in endometriosis.

#### Data Harmonisation and Standardisation

Data harmonisation and standardisation remain major barriers to the clinical translation of AI-based endometriosis diagnostics. Imaging models require consistent acquisition protocols, anatomical definitions, and annotation standards, particularly for TVUS sliding-sign assessment and MRI-based detection of pouch of Douglas obliteration [22,23,37]. Similarly molecular, and multi-omics models are affected by differences in sample type, sequencing or assay platform, pre-processing pipeline, feature selection and batch-effect correction, which may limit reproducibility across centres [24,25,27,28,31]. Future studies should therefore prioritise standardised data collection, transparent pre-processing methods and multicentre validation to ensure that AI models remain robust across different populations, platforms, and clinical settings.

Real-world implementation also requires attention to population diversity, device variability and regulatory oversight. Models developed in specialist centres or homogeneous cohorts may not perform equally across different populations, ethnic groups, symptom profiles or healthcare settings [19,20,22]. Imaging-based AI tools may also be affected by differences in ultrasound or MRI equipment, acquisition protocols, operator expertise and image quality, which can reduce reproducibility when applied outside the development environment [22,23,37]. Before clinical deployment, AI systems should therefore undergo local validation, regulatory review, continuous performance monitoring and clear governance regarding accountability, data privacy and safe clinician oversight [9,11,12].

### 4.3. Explainability, Workflow Integration and Clinician Trust

In addition to accuracy and validation, real-world implementation requires careful consideration of model interpretability, integration into existing gynaecological workflows, and clinician trust [11]. This is particularly important in imaging and molecular diagnostics, where algorithmic outputs may be difficult to verify without a transparent explanation. In imaging-based models, explainable AI approaches, including visual heatmaps or saliency-based localisation, may help demonstrate which anatomical regions or sonographic features contributed to the model output [22,23]. In molecular and multi-omics models, feature-importance approaches may help identify which genes, miRNAs, cytokines or biomarker patterns drive classification [24,25,27]. Such transparency is essential to support clinical accountability, reduce inappropriate reliance on automated outputs, and promote acceptance of AI as a decision-support tool rather than a standalone diagnostic replacement [11].

### 4.4. Future Directions

To provide a structured pathway for future work, the opportunities identified in this review can be organised into short-, medium-, and long-term priorities. In the short term, emphasis should be placed on improving dataset quality, standardising imaging protocols, and ensuring external validation of existing models across diverse populations. Medium-term priorities should focus on integrating explainable AI tools into clinical workflows, establishing regulatory and ethical frameworks, and developing multicentre collaborations to enhance generalisability. In the longer term, the aim should be to combine multimodal data, including imaging, biomarkers, molecular signatures, and NLP/LLM outputs, into unified diagnostic platforms supported by robust clinical trials. This staged approach will help translate current proof-of-concept advances into safe, reliable, and widely adopted clinical tools.

## 5. Proposed AI-Enabled Diagnostic Workflow

**Figure 2:**
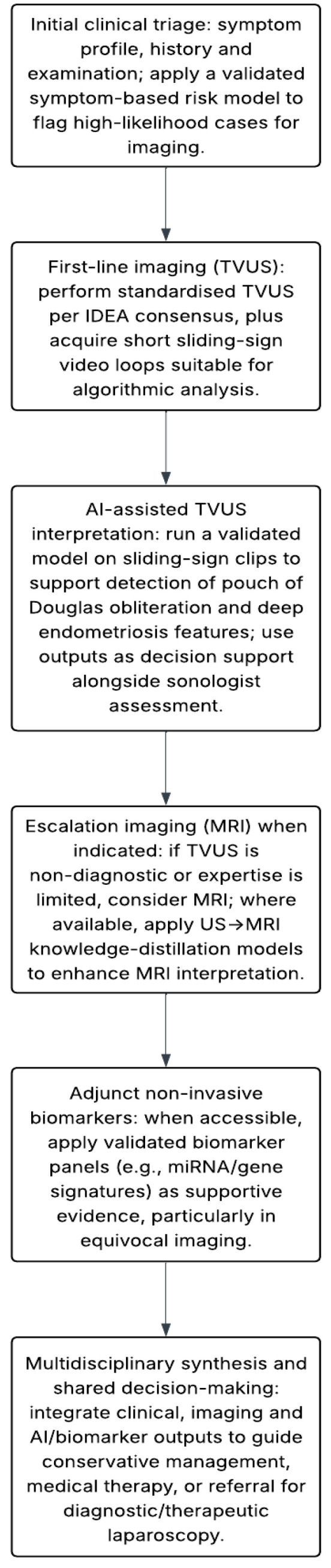
Proposed AI-enabled diagnostic workflow for endometriosis, illustrating how symptom-based triage, standardised TVUS, AI-assisted imaging interpretation, MRI escalation, non-invasive biomarkers and multidisciplinary decision-making may support existing diagnostic pathways.

A stepwise AI-enabled diagnostic workflow summarising how AI can augment current practice is proposed. For clinical implementation, the proposed AI-enabled workflow should be integrated as a decision-support pathway rather than a standalone diagnostic system. This would require regulatory approval, local validation within existing gynaecological services, standardised TVUS/MRI acquisition protocols, secure data infrastructure, and clear governance regarding data privacy and clinical accountability [11,22,23,37]. Clinician training would also be essential to ensure correct image acquisition, interpretation of AI outputs and appropriate communication of uncertainty to patients [22,37]. Cost considerations, including software integration, equipment compatibility, staff training and maintenance, should be evaluated before wider adoption, particularly in settings with limited specialist imaging expertise [9,36] (Figure 2).

## 6. Conclusions

AI has the potential to assist in identifying previously unrecognised patterns, which could enhance diagnostic pathways as evidence and validation grow. Recent advancements in biomarkers, for instance, BCL6 expression, programmed cell-death-related genes and persistent organic pollutants, are expanding the non-invasive diagnostic tools. In parallel, AI has shown significant potential in the clinical approach to endometriosis, ranging from symptom-based prediction to assessment of POD obliteration using TVUS. Studies have exhibited its potential, and it could potentially serve as a valuable tool in research and diagnostics, improving the delay, and the significant negative impact it holds onto women.

These innovations show a clear path towards earlier diagnosis and individualised risk stratification; however, further studies are required to fully unveil the true nature of the disease. Even though AI-driven methods have not been subject to randomised controlled clinical trials, their potential to transform healthcare is evident. As the world of research continues to expand, AI holds the power to recognise previously unidentified patterns.

Despite all the advantages, AI carries common pitfalls, such as overfitting, small sample sizes, lack of external validation and lower AUC scores. As datasets and technologies are constantly evolving, it is only a matter of time before AI can combat these limitations and refine itself. However, it is imperative that strict regulations are put in place before AI is translated into everyday practice. A multidisciplinary framework uniting molecular diagnostics with AI algorithms may ultimately improve the quality of life in women.

## Data Availability

No new data were created or analyzed in this study. Data sharing is not applicable to this article.

## Supplementary Materials

The following supporting information can be downloaded at: https://www.mdpi.com/article/10.3390/diagnostics16121823/s1, File S1: PRISMA checklist [52].

## Abbreviations

The following abbreviations are used in this manuscript:

AI	Artificial Intelligence
CPP	Chronic Pelvic Pain
USA	United States of America
UK	United Kingdom
ML	Machine Learning
DL	Deep Learning
NLP	Natural Language Processing
LLM	Large Language Model
GPT	Generative Pre-Trained Transformer
GPT-4	Generative Pre-Trained Transformer 4
ESHRE	European Society of Human Reproduction and Embryology
AUC	Area Under the Curve
GSWH	Global Study of Women’s Health
WHSS	Women’s Health Symptom Survey
CRESCENDO	Creating Prediction Model to Predict Surgical Success in Endometriosis
IDEA	International Deep Endometriosis Analysis
TVUS	Transvaginal Ultrasound
POD	Pouch of Douglas
MRI	Magnetic Resonance Imaging
US	Ultrasound
IHC	Immunohistochemistry
CD10	Cluster of Differentiation 10
DIE	Deeply Infiltrating Endometriosis
DAPI	4′,6-diamidino-2-phenylindole
SMA	Smooth Muscle Actin
BCL6	B-cell Lymphoma 6
GC	Germinal Centre
TFH	T Follicular Helper
HSCORE	Histological Score
RNA	Ribonucleic Acid
MAPK1	Mitogen-Activated Protein Kinase 1
PCD	Programmed Cell Death
TNFSF12	Tumour Necrosis Factor Superfamily Member 12
PDK2	Pyruvate Dehydrogenase Kinase 2
ACOG	American College of Obstetricians and Gynecologists
